# Treatment of Perinatal Depression and Correlates of Treatment Response Among Pregnant Women Living with HIV in Uganda

**DOI:** 10.1007/s10995-023-03741-1

**Published:** 2023-06-24

**Authors:** Laura J. Faherty, Violet Gwokyalya, Akena Dickens, Ryan McBain, Vicky Ngo, Janet Nakigudde, Juliet Nakku, Barbara Mukasa, Jolly Beyeza-Kashesya, Rhoda K. Wanyenze, Glenn J. Wagner

**Affiliations:** 1 RAND Corporation, 20 Park Plaza, Suite 920, Boston, MA 02128 USA; 2 Department of Pediatrics, Maine Medical Center, 22 Bramhall St, Portland, ME 04102 USA; 3 Makerere University, 7062 University Rd, Kampala, Uganda; 4 Graduate School of Public Health and Health Policy, City University of New York, 205 E 42nd St, New York, NY 10017 USA; 5 Mildmay Uganda, P.O Box 24985, Kampala, Uganda; 6 RAND Corporation, 1776 Main St, Santa Monica, CA 90407 USA

**Keywords:** HIV, PMTCT, Uganda, Depression, Antidepressants, Perinatal, Problem Solving Therapy

## Abstract

**Introduction:**

Perinatal depression is common among women living with HIV, but depression care is limited in low-resource settings. We examined (1) characteristics of women receiving Problem Solving Therapy (PST) versus antidepressant therapy (ADT), (2) treatment response by modality, and (3) correlates of treatment response.

**Methods:**

This analysis used data from 191 Ugandan women in the intervention arm of a cluster randomized controlled trial of task-shifted, stepped-care depression treatment for pregnant women living with HIV (PWLWH). Treatment response was defined as scoring < 5 on the nine-item Patient Health Questionnaire (PHQ-9). Bivariate analysis and multivariable logistic regression were used to examine characteristics of women by treatment group and correlates of treatment response.

**Results:**

Of 134 participants with depression, 129 (96%) were treated: 84 (65%) received PST and 45 (35%) received ADT. Severe depression at treatment initiation was more common in those receiving ADT (28.9% versus 4.8%, Fischer’s Exact Test < 0.001). Treatment response was higher for PST (70/84; 83.3%) than ADT (30/45; 66.7%; p = .03). ADT side effects were rare and minor; no infants had serious congenital defects. Of 22 participants (19%) who did not respond to treatment, only five received intensified management. Social support and interpersonal violence were associated with treatment response (adjusted odds ratio, [aOR] = 3.06, 95% CI = 1.08–8.66 and aOR = 0.64, 95% CI = 0.44–0.93).

**Discussion:**

Both depression treatment modalities yielded high response rates in Ugandan PWLWH; ADT was well-tolerated. Our results highlight a need to build capacity to implement the stepped-care protocol for non-responders and screen for social support and interpersonal violence.

**Supplementary Information:**

The online version contains supplementary material available at 10.1007/s10995-023-03741-1.

## Significance

*What is Already Known on this Subject?*Prior research has demonstrated the feasibility and effectiveness of delivering collaborative care for depression in low-resource settings including Uganda.

*What this Study adds?*Both Problem-Solving Therapy and antidepressant therapy yielded high treatment response rates among pregnant women living with HIV in Uganda who met criteria for depression, and antidepressant therapy was well tolerated. This study highlights a need to build capacity to implement the task-shifted stepped-care protocol for women whose depression does not respond to initial treatment, and to screen for social support and interpersonal violence.

**Supplementary Information:**

The online version contains supplementary material available at 10.1007/s10995-023-03741-1.

## Introduction

Perinatal depression is common among pregnant women living with HIV (PWLWH), with approximately one in three meeting criteria for clinical depression in sub-Saharan Africa (SSA) (Sowa, et al., 2015), and up to half experiencing depressive symptoms during pregnancy or postpartum. Perinatal depression can cause negative outcomes for the mother-infant dyad. Studies of PWLWH have shown that depression is associated with less adherence to protocols for prevention of mother-to-child transmission (PMTCT) as well as more rapid HIV disease progression and death (Antelman, et al., 2007; Mayston, et al., 2012; Nakimuli-Mpungu, et al., 2012; Starace, et al., 2002). Perinatal depression can adversely affect birth outcomes, maternal-infant attachment, and child development (Grote, et al., 2010; Pearson, et al., 2013; Surkan, et al., 2011; Tronick et al. 2009).

While evidence-based treatments for perinatal depression exist (Yonkers, et al., 2009), it is rarely screened for, diagnosed, and treated in Uganda. Mental health professionals are scarce (Kigozi, et al., 2010), and stigma prevents people from seeking care and disclosing symptoms (Kitafuna, 2022). Prior research has demonstrated the feasibility and effectiveness of delivering collaborative care for depression in low-resource settings (Nyamayaro, et al., 2020; Patel, et al., 2007, 2010), including Uganda (Wagner, et al., 2017), and for underserved populations in the United States (LaRocco-Cockburn, et al., 2013). However, its effectiveness has not been evaluated in the context of treating depression among PWLWH in SSA.

To contribute to this body of evidence, we are conducting a cluster randomized controlled trial (Maternal Depression Treatment in HIV, M-DEPTH). Drawing on evidence-based collaborative care models for depression in low-resource settings (Patel, et al., 2007), we are implementing a stepped-care approach to offering psychological and pharmacological treatment options for PWLWH in Uganda who meet criteria for depression. As part of the larger study evaluating the effects of depression treatment with Problem Solving Therapy (PST) or antidepressant therapy (ADT) on PMTCT care adherence and outcomes, we examined (1) characteristics of participants who received PST versus ADT, (2) response to depression treatment by treatment modality, and (3) correlates of treatment response. Our prior work documented the effectiveness of the treatment model for reducing depressive symptoms in PWLWH, with the intervention group being 80% less likely to be depressed than controls at follow-up (Wagner, et al., 2023). To help health care providers in low resource settings counsel their pregnant patients on the risks and benefits of depression treatment options (Molenaar, et al., 2018), this paper provides a more in-depth analysis of the two treatment modalities. We hypothesized that both PST and ADT would have high treatment response rates among Ugandan PWLWH and that ADT would be well-tolerated.

## Methods

### Study Design

Details of this prospective, multi-site cluster randomized controlled trial, which follows CONSORT guidelines, can be found elsewhere (Wagner, et al., 2019). This ongoing trial evaluates the effects of task-shifted, evidence-based depression care, relative to usual care, on PMTCT adherence and maternal-infant health outcomes. The trial is being conducted at eight public antenatal care clinics in Uganda, four randomly assigned to administer usual care and four to usual care plus the depression care model. This paper focuses on participants in the intervention arm. Participants are enrolled during pregnancy and followed through pregnancy completion until 18 months postpartum (or six months after premature pregnancy loss or termination). Participants are assessed at baseline, and at two, six, 12, and 18 months post-pregnancy completion. Data collection is complete through the six-month post-pregnancy assessment, and these data are used for this analysis, as this is sufficient time post-partum for monitoring response to depression treatment. The study protocol was approved by the corresponding author’s institutional review board.

### Study Participants

To be eligible for this study, adult PWLWH had to score > 0 on the PHQ-2, which is administered by peer mothers at all ANC visits. Potential participants were further evaluated by a nurse who applied the following inclusion criteria: (1) gestation period of 32 weeks or less, (2) medically stable without opportunistic infections and on antiretroviral therapy (ART) for at least 4 weeks, (3) age 18 years or older, and (4) scored > 4 on the nurse-administered 9-item Patient Health Questionnaire (PHQ-9) (Kroenke, et al., 2001). This threshold was selected to identify women with at least minor depressive symptoms. Women were excluded if already receiving mental health treatment or at elevated risk of suicide. Participants provided written consent, and recruitment occurred between July 2019 and January 2021.

### M-DEPTH Depression Care Model

The M-DEPTH Depression Care model consisted of (1) *depression screening* by peer mothers using the PHQ-2; (2) *evaluation of treatment eligibility* for those who screened positive by the nurse using the PHQ-9, and who met study inclusion criteria; (3) *depression psychoeducation and recommendation of treatment modality* by the nurse (World Health Organization, 2016), consisting of PST for moderate depression (PHQ-9 scores of 10–19) and ADT for severe depression (PHQ-9 > 19); and (4) *provision of treatment* selected by the patient, by peer mothers (PST) and nurses (ADT), supervised by mental health specialists.

#### Problem Solving Therapy (PST)

PST is a cognitive-behavioral intervention that teaches patients to use adaptive problem solving and systematically apply four skills: defining the problem, generating solutions, selecting solutions, and implementing/evaluating those solutions (Bell et al. 2009; Malouff, et al., 2007). Peer mothers received training to implement manualized individual PST over three biweekly core sessions, followed by up to four optional monthly sessions, for a maximum of seven sessions.

#### Antidepressant Therapy (ADT)

ADT was administered and monitored by nurses, beginning in the second trimester to balance benefits with risks to the developing fetus. Fluoxetine, a selective serotonin reuptake inhibitor (SSRI), at a starting daily dose of 20 mg was the first-line medication, and imipramine, a tricyclic antidepressant, at a starting daily dose of 50 mg (increasing to 75 mg after the first week) was the second-line option. At monthly follow-up visits, measures of treatment response and assessment of side effects guided changes to dosage or medication. Participants receiving ADT were instructed to monitor their infant for signs of poor neonatal adaptation after birth. When the participant’s PHQ-9 was < 5 for six months, and she was at least six months post-pregnancy completion, medication discontinuation was considered.

If a patient’s PHQ-9 scores indicated she was not responding to treatment or was experiencing a relapse of symptoms after initial response, depression management was to be “stepped-up” in intensity. This stepped-care approach could include adding the other modality (e.g., ADT if receiving PST or vice versa), switching from PST to ADT, or increasing ADT dosage.

### Measures

Assessments included surveys, laboratory viral load testing, pharmacy data, and data abstracted from medical charts and a Depression Care Registry maintained by peer mothers and nurses. Survey measures were interviewer-administered; those that had not been translated into Luganda, the local language, during our prior research were translated using standard translation and back-translation methods.

Response to depression treatment is the focus of this analysis. At baseline and two months post-pregnancy completion, the PHQ-9, which has been used successfully in rural Uganda (Nakku, et al., 2016; Kaggwa, et al., 2022), was administered as part of the study survey. In addition, the PHQ-9 was administered at each clinic visit for participants in the intervention arm through six months postpartum. The scale’s nine items correspond to the nine symptoms assessed in the depression module of the Diagnostic Statistical Manual of Mental Disorders (American Psychiatric Association, 1994). PHQ-9 scores above 9 (range: 0 to 27) have been shown to correspond with major depressive disorder (Kroenke, et al., 2001), and this cutoff was used to represent clinical depression in this study. We defined treatment response as a PHQ-9 score < 5 at any point during treatment.

Survey measures included sociodemographic characteristics (age and any secondary education); pregnancy-related characteristics (gestation length, pregnancy outcome, whether pregnancy was planned); health functioning and quality of life; depression treatment-related factors (PHQ-9 at treatment initiation, weeks on treatment); relationship factors (e.g., relationship status, partner knowledge of patient’s HIV status, partner support and involvement in the pregnancy; and experience with interpersonal violence [IPV]); experience of trauma (childhood or recent); PMTCT knowledge and attitudes; and problem solving orientation (positive, negative, or avoidant). Surveys also asked about any ADT side effects, degree to which they interfered with daily life, and infant signs of poor neonatal adaptation (i.e., jitteriness, respiratory distress). Mothers were asked if they sought medical care for these signs.

Data related to HIV diagnosis and care (e.g., time since diagnosis, whether ART was started during current pregnancy) and whether the infant had any serious congenital conditions were abstracted from the patient’s clinical chart. An HIV viral load assay was collected at baseline and two months post-pregnancy completion, and percent of prescribed ART pills taken was calculated using pharmacy refill data.

The Online Supplement provides additional detail on these measures.

### Statistical Analysis

We used descriptive statistics to examine depression treatment response by modality (PST or ADT) and the presence of side effects. We conducted bivariate analyses to examine differences in patient characteristics between those treated with PST versus ADT, and those whose depression did or did not respond to treatment. We used chi-squared tests for categorical variables and two-tailed independent t-tests for continuous variables, with an *a priori* significance level of *P* < .05.

We used multivariable logistic regression models, estimated using SAS Proc Surveylogistic, to further examine predictors of treatment response using variables found to be significant correlated with response in bivariate analyses. These models were adjusted for baseline demographics (age, any secondary education, relationship status), weeks on depression treatment, and study site. Analyses were conducted using SAS version 9.2 (SAS Institute, Cary, NC).

## Results

### Sample Characteristics

The intervention arm consisted of 191 PWLWH (out of 391 PWLWH enrolled in the trial overall). Baseline characteristics of those in the intervention arm included mean age of 27.3 (SD = 5.7) years, 41% had any secondary education, 81% were in a committed relationship, mean gestation was 21.3 weeks (SD = 6.4), 22% were newly diagnosed with HIV, and 57% had an undetectable HIV viral load.

Of the 191 PWLWH, 122 (63.8%) met criteria for clinical depression (PHQ-9 > 9) at baseline and 117 (95.9%) of those received depression treatment. Of the five participants who were not started on treatment at baseline, two declined treatment, two were lost to follow-up or transferred their care elsewhere, and one had a rapid decrease in her PHQ-9 score before initiating treatment. An additional 12 participants with mild depressive symptoms at baseline, (PHQ-9 between 5 and 9), reported increased depression (PHQ-9 > 9) in the weeks following study enrollment (range: 2–13 weeks after enrollment), and were initiated on treatment. Therefore, a total of 129 (96.2%) of the 134 participants who met criteria for clinical depression either at baseline or after study enrollment received depression treatment, 84 (65.1%) of whom began PST and 45 (34.8%) of whom began ADT.

Depressive symptoms at treatment initiation were more severe among those treated with ADT compared to PST, based on mean (SD) PHQ-9 ([16.4 (4.1) vs. 13.3 (3.2); p < .001] and the proportion of participants with severe depression (PHQ-9 > 19; 28.9% vs. 4.8%; Fischer’s Exact Test < 0.001). Participants initiated on ADT were younger [mean (SD) age = 25.2 (5.4) vs. 28.4 (5.3); p < .001] and had higher HIV viral loads at baseline [mean (SD) log_10_ viral load = 1.5 (1.7) vs. 0.8 (1.2); p = .006]. The two subgroups did not differ on other sociodemographic or HIV disease characteristics, nor on gestation length.

### Treatment Effects on PHQ-9 Scores

#### Overall

Of the 129 participants who received depression treatment with PST or ADT, 100 (77.5%) responded to treatment (PHQ-9 < 5) at some point during the study and remained in remission at the conclusion of treatment (or at study completion for those who remained on ADT).

PHQ-9 scores at the start of treatment were significantly higher among participants who did not respond to treatment than among those who did [15.9 (SD = 4.0) vs. 14.0 (SD = 3.7), p = .018], as was the proportion of participants with severe depression (27.6% vs. 9.0%, p = .009). The response rate was significantly higher among those treated with PST (70/84; 83.3%) compared to those treated with ADT (30/45; 66.7%) (p = .031).

#### By Treatment Modality: Problem Solving Therapy

Among the 84 participants who were treated with PST, the mean number of sessions attended was 5.9 (SD = 2.1; median = 6); 78 (92.9%) completed the three core sessions, and 74 of those 78 (94.9%) completed additional sessions. A total of 74 women (88.1%) responded to PST at some point in the study. Treatment response was achieved after a mean of 13.1 weeks (SD = 7.6), and the mean PHQ-9 at the conclusion of treatment was 1.9 (SD = 2.5). Among the 74 who responded, 17 (23.0%) experienced a relapse of depression, of whom 13 had their depression return to a state of remission [PHQ-9 < 5; mean (SD) PHQ-9 = 3.4 (2.8)] by the end of treatment. Therefore, 70 (83.3%) participants responded to PST overall.

Of the 10 (11.9%) whose depression never responded to PST, 4 prematurely discontinued treatment. ADT was added to PST for only one of the 10 participants who did not respond, but not for any of the 17 who experienced a relapse.

#### By Treatment Modality: Antidepressant Therapy

Among the 45 participants who received ADT, 43 (95.6%) were prescribed fluoxetine and 2 (4.4%) received imipramine; only one woman had a dosage increase of her medication. At this point in the ongoing study, mean number of months of ADT is 9.3 (SD = 4.8; range: 2 weeks to 25 months). Thirty-three women (73.3%) responded to ADT after a mean of 19.2 weeks (SD = 14.0), and the mean PHQ-9 at the conclusion of either treatment or study participation was 1.9 (SD = 2.0).

Among the 33 participants who responded to ADT,13 (39.4%) experienced worsening of their depression after initial response, including three who began receiving PST in addition to ADT. Among these 13, 10 (78.6%) (including the three who received augmentative PST) had their depression return to a state of remission at the end of treatment or the study. Therefore, 30 (66.7%) participants responded to ADT overall.

Of the 12 (26.7%) whose depression never responded to ADT, 4 prematurely discontinued treatment, 3 added PST (two of which then responded to treatment), and 1 was switched to PST from ADT.

### Correlates of Treatment Response

Table 1 lists the bivariate correlates of treatment response. IPV, any childhood trauma, and number of recent traumas were all negatively associated with treatment response. General social support, health-related quality of life, attitudes (but not knowledge) about prevention of maternal-to-child transmission of HIV, and positive problem-solving orientation were positively associated with treatment response. No sociodemographic or HIV-related medical characteristics were associated with depression treatment response, nor were pregnancy characteristics (Table 1).

**Table 1:** Characteristics of participants who did and did not respond to depression treatment^a^

Characteristics	Responded (n = 100)	Did not respond (n = 29)	
	Mean (SD) or percentage	Mean (SD) or percentage	p/FET
Sociodemographic factors			
Age	27.5 (5.5)	26.7 (5.8)	0.468
Any secondary education	34.3%	48.3%	0.173
HIV- and pregnancy-related factors			
Length of time since HIV diagnosis (months)	47.8 (45.8)	43.5 (44.7)	0.656
Started antiretroviral therapy during current pregnancy	17.2%	21.2%	0.640
Undetectable viral load	62.1%	58.6%	0.737
Length of gestation	21.1 (6.6)	21.3 (6.2)	0.830
Successful delivery of child	93.9%	92.9%	0.561
Quality of life and depression-related factors			
**Health functioning QOL**	**52.9 (12.3)**	**46.6 (12.7)**	**0.018**
**PHQ-9 at start of treatment**	**14.0 (3.7)**	**15.9 (4.0)**	**0.018**
**Severe depression (PHQ-9 > = 20)**	**9.0%**	**27.6%**	**0.009**
Weeks on depression treatment at time of postpartum visit	14.9 (9.8)	17.9 (8.0)	0.108
Relationship factors			
**General social support**	**2.3 (0.7)**	**1.9 (0.5)**	**< 0.001**
In a committed relationship	72.4%	83.8%	0.166
Partner knows respondent’s HIV status	51.7%	70.7%	0.057
Pregnancy was planned	42.4%	41.4%	0.920
Partner support for the pregnancy	2.4 (1.2)	2.6 (1.0)	0.290
Partner involvement in ANC/PMTCT	3.6 (2.7)	2.6 (2.4)	0.067
**IPV (sum of all types of IPV)**	**3.4 (1.8)**	**4.7 (1.8)**	**0.006**
IPV- controlling type	3.0 (1.2)	2.4 (1.2)	0.044
IPV – physical type	0.9 (0.8)	0.4 (0.6)	0.001
IPV – sexual type	0.3 (0.5)	0.3 (0.5)	0.616
Experience of trauma			
**Any childhood trauma**	**80.8%**	**96.6%**	**0.043**
**Number of recent traumas**	**2.0 (1.9)**	**2.9 (1.8)**	**0.031**
Knowledge, attitudes, and problem-solving orientation			
PMTCT knowledge	4.1 (1.2)	3.5 (1.6)	0.098
**PMTCT attitudes**	**4.2 (0.3)**	**4.0 (0.1)**	**0.008**
Negative problem-solving	10.8 (4.2)	11.8 (5.2)	0.279
**Positive problem-solving**	**14.8 (4.0)**	**12.5 (4.3)**	**0.008**
Avoidant problem-solving	5.3 (4.3)	7.0 (5.1)	0.078

^a^ Predictors found to be statistically significant at the P < .05 level are bolded

Abbreviations: FET = Fischer’s Exact Test; ANC = antenatal care; IPV = interpersonal violence; HIV = human immunodeficiency virus; Q = quality of life; PHQ-9 = patient health questionnaire = 9

In multivariable analyses, only two factors were associated with treatment response: general social support was associated with higher odds of responding to depression treatment (adjusted odds ratio, [aOR] = 3.06, 95% CI = 1.08–8.66), and IPV was associated with lower odds of treatment response (aOR = 0.64, 95% CI = 0.44–0.93) (Table 2).

**Table 2:** Regression models examining predictors of response to depression treatment

Characteristics	Odds Ratio (95% CI)
**General social support**	**3.06 (1.08–8.66)**
**Interpersonal violence**	**0.64 (0.44–0.93)**
Any childhood trauma	0.00 (0.00–0.00)
Number of recent traumas	0.86 (0.62–1.19)
Health functioning QOL	1.03 (0.97–1.09)
PMTCT attitudes	3.35 (0.15–75.63)
Positive problem solving	1.14 (0.96–1.36)
PHQ-9 at start of treatment	1.00 (0.84–1.19)

Abbreviations: CI = confidence interval; QOL = quality of life; PMTCT: prevention of mother-to-child transmission; PHQ-9: patient health questionnaire-9

Characteristics found to be statistically significant are bolded

### Side Effects of Antidepressant Therapy

Of the 45 participants who received ADT, 40 (88.9%) provided data about side effects at the postpartum assessment. Nine participants (22.5%) reported side effects, of whom five said they were “somewhat” bothersome and 1 said they interfered “more than a little” with daily life. When asked about physical signs in their infant that could have been related to *in utero* SSRI exposure, 29 participants (72.5%) reported that their infant had trouble breathing, 7 (17.5%) reported jitteriness, and three (7.5%) reported irritability/excessive crying. However, only two of the 29 participants (6.9%) reporting bringing the infant to the clinic because of these physical signs. No infants were born with serious birth defects or major medical conditions (data not shown).

## Discussion

The treatment response rate among Ugandan PWLWH receiving depression care using an evidence-based collaborative care model was high overall, consistent with other studies, (Patel, et al., 2007, 2010; Nyamayaro, et al., 2020) and higher among participants receiving PST versus ADT. However, fewer participants in the former group had severe depression. While most participants in the intervention arm of this trial responded to depression treatment, our results highlight a need to build capacity to implement the stepped-care model for those that do not respond to treatment. Social support and IPV were found to be associated with treatment response in the positive and negative direction, respectively.

Nearly all (96%) participants who were identified as having depression received treatment, and over three-quarters of treated participants responded to PST or ADT. This encouraging result is consistent with existing literature on the effectiveness of psychological interventions such as PST for people living with HIV (van Luenen, et al., 2018), including the use of lay health workers to deliver evidence-based mental health care to people living with HIV (Nakimuli-Mpungu, et al., 2020), but extends it to the population of pregnant and postpartum participants. Our study is also among the first to document the effectiveness of ADT in PWLWH in Uganda or the larger region of sub-Saharan Africa for alleviating perinatal depression. Furthermore, the low rate of side effects for both the mother and the infant, the absence of serious birth defects or major medical conditions in the infants of mothers treated with ADT during pregnancy, and the fact that some participants with less severe depression chose ADT despite being recommended PST per protocol, demonstrate that ADT was well-tolerated and acceptable in this population.

The finding that the treatment response rate was higher among those receiving PST (83.3%), compared to those receiving ADT (66.7%), deserves future study. One potential explanation is that more participants had severe depression symptoms at the start of treatment in the ADT group (29%) than the PST group (5%). Both groups were receiving support through the Family Support Group program (see Online Supplement for description of this multi-session group program to support PMTCT and pregnancy management instituted by the Ugandan Ministry of Health) as part of usual care, but perhaps the PST sessions, which could be tailored to address the specific problems facing the patient, better equipped patients with skills to manage their depression and related stressors (Malouff, et al., 2007; van Luenen, et al., 2018). In contrast, those receiving ADT might not have felt as empowered to address their daily challenges.

For participants who did not respond to their initial treatment modality, providers made few changes to management (i.e., they were not “stepped up” in therapy intensity). In particular, of the 22 participants (10 on PST, 12 on ADT) who never responded to treatment, only five were switched (n = 1) or had another treatment modality added (n = 4) to improve response, although eight of these participants (four on each modality) prematurely discontinued treatment, thus having less time to experience a change in treatment. While PST could be intensified by adding sessions, there were no dosage increases among participants receiving ADT. These results suggest a need to strengthen the provision of supportive supervision and consider refresher trainings for providers, as prior studies have documented the importance of these supports for maintaining skills over time and ensuring fidelity to treatment protocols (Murray, et al., 2011; Nakku, et al., 2021).

Limited research exists on factors influencing response to depression treatment in this population. However, our observations that greater social support and less intimate partner violence were associated with greater likelihood of treatment response are consistent with a prior Ugandan study (Atuhaire, et al., 2021) and a systematic review conducted outside of SSA (Lancaster, et al., 2010). More research exists on factors associated with development of depression than response to treatment. For instance, studies from rural Uganda (Kakyo, et al., 2012) and South Africa (Stellenberg et al. 2015) found that relationships with male partners was a major contributor to postpartum depression, an association that was not present in our study. In bivariate analyses, exposure to trauma and lower physical functioning and quality of life were associated with non-response to depression treatment. While these factors did not remain significant in multivariable analyses, they warrant further study.

This study has limitations. We do not have data on decision-making around selection of initial depression treatment modality, nor fidelity to interventions (e.g., adherence to ADT, engagement in PST sessions), which could affect treatment response. In addition, due to sample size, we could not examine differential effectiveness of treatment modalities by depression severity. Lastly, the treatment efficacy data presented reflect response to open treatment; the comparison to usual care is presented elsewhere in the context of intervention effects on PMTCT outcomes, for which depression outcomes were a mediator (Wagner et al., submitted).

These results highlight that non-mental health experts, specifically nurses and lay persons, can effectively identify depression and implement both psychological and pharmacologic evidence-based depression treatment with training and supervision by specialists. Nearly all participants who needed treatment received the treatment of their choice (predominantly PST), and nearly three-quarters responded to treatment, which is at least equivalent to response rates in the literature. Our data also demonstrate the importance of screening for social support and IPV to identify PWLWH at risk for or in need of treatment for perinatal depression. These findings will help inform efforts to strengthen the provision of evidence-based, individualized perinatal depression care to PWLWH in Uganda and other low resource settings.

### Electronic Supplementary Material

Below is the link to the electronic supplementary material.

Supplementary Material 1

## Data Availability

Study data may be made available upon request.

## Code Availability

N/A.

## References

[CR1] American Psychiatric Association (1994). Diagnostic Statistical Manual of Mental Disorders (Washington, DC).

[CR2] Antelman G, Kaaya S, Wei R, Mbwambo J, Msamanga GI, Wafaie W, Fawzi (2007). Depressive symptoms increase risk of HIV disease progression and mortality among women in Tanzania. Journal of Acquired Immune Deficiency Syndromes.

[CR3] Atuhaire C, Rukundo GZ, Nambozi G, Ngonzi J, Atwine D, Cumber SN (2021). Prevalence of postpartum depression and associated factors among women in Mbarara and Rwampara districts of south-western Uganda. BMC Pregnancy and Childbirth.

[CR4] Bell AC, D’Zurilla TJ (2009). Problem-solving therapy for depression: A meta-analysis. Clininical Psychology Reviews.

[CR5] Grote NK, Jeffrey A, Bridge, Amelia R, Gavin JL, Melville S, Wayne JK (2010). A meta-analysis of depression during pregnancy and the risk of preterm birth, low birth weight, and intrauterine growth restriction. Archives of General Psychiatry.

[CR6] Kaggwa MM, Najjuka SM, Ashaba S, Mamun MA (2022). Psychometrics of the Patient Health Questionnaire (PHQ-9) in Uganda: A systematic review. Frontiers in Psychiatry.

[CR7] Kakyo TA, Muliira JK, Mbalinda SN, Kizza IB, Muliira RS (2012). Factors associated with depressive symptoms among postpartum mothers in a rural district in Uganda. Midwifery.

[CR8] Kigozi, F., Ssebunnya, J., Kizza, D., Cooper, S., Ndyanabangi, S. (2010). Health the Mentt al. An overview of Uganda’s mental health care system: results from an assessment using the world health organization’s assessment instrument for mental health systems (WHO-AIMS), International Journal of Mental Health Systems, 4(1):1. 10.1186/1752-4458-4-1 PMC2831025 20180979

[CR9] Kitafuna KB (2022). A critical overview of mental health-related beliefs, services and systems in Uganda and recent activist and legal challenges. Community Mental Health Journal.

[CR10] Kroenke K, Spitzer RL, JBW Williams (2001). The PHQ-9. Journal of General Internal Medicine.

[CR11] Lancaster CA, Gold KJ, Flynn HA, Yoo H, Marcus SM, Davis MM (2010). Risk factors for depressive symptoms during pregnancy: A systematic review. American Journal of Obstetrics & Gynecology.

[CR12] LaRocco-Cockburn A, Reed SD, Melville J, Croicu C, Russo JE, Inspektor M (2013). Improving depression treatment for women: Integrating a collaborative care depression intervention into OB-GYN care. Contemporary Clinical Trials.

[CR13] Malouff JM, Thorsteinsson EB, Schutte NS (2007). The efficacy of problem solving therapy in reducing mental and physical health problems: A meta-analysis. Clininical Psychology Reviews.

[CR14] Mayston R, Kinyanda E, Chishinga N, Prince M, Vikram Patel (2012). Mental disorder and the outcome of HIV/AIDS in low-income and middle-income countries: A systematic review. Aids (London, England).

[CR15] Molenaar NM, Kamperman AM, Boyce P, Bergink V (2018). Guidelines on treatment of perinatal depression with antidepressants: An international review. Australian And New Zealand Journal Of Psychiatry.

[CR16] Murray LK, Dorsey S, Bolton P, Jordans MJ, Rahman A, Bass J (2011). Building capacity in mental health interventions in low resource countries: An apprenticeship model for training local providers. International Journal of Mental Health Systems.

[CR17] Nakimuli-Mpungu E, Bass JK, Alexandre P, Mills EJ, Musisi S, Ram M (2012). Depression, alcohol use and adherence to antiretroviral therapy in sub-saharan Africa: A systematic review. AIDS and Behavior.

[CR18] Nakimuli-Mpungu E, Musisi S, Wamala K, Okello J, Ndyanabangi S, Birungi J (2020). Effectiveness and cost-effectiveness of group support psychotherapy delivered by trained lay health workers for depression treatment among people with HIV in Uganda: A cluster-randomised trial. The Lancet Global Health.

[CR19] Nakku JEM, Nalwadda O, Garman E, Honikman S, Hanlon C, Kigozi F (2021). Group problem solving therapy for perinatal depression in primary health care settings in rural Uganda: An intervention cohort study. BMC Pregnancy and Childbirth.

[CR20] Nakku JEM, Rathod SD, Kizza D, Breuer E, Mutyaba K, Baron EC (2016). Validity and diagnostic accuracy of the Luganda version of the 9-item and 2-item Patient Health Questionnaire for detecting major depressive disorder in rural Uganda. Global Mental Health (Cambridge).

[CR21] Nyamayaro P, Bere T, Magidson JF, Simms V, Conall O’Cleirigh D, Chibanda (2020). A task-shifting problem-solving therapy intervention for depression and barriers to antiretroviral therapy adherence for people living with HIV in Zimbabwe: Case series. Cognitive and Behavioral Practice.

[CR22] Patel V, Araya R, Chatterjee S, Chisholm D, Cohen A, De Silva M (2007). Treatment and prevention of mental disorders in low-income and middle-income countries. The Lancet.

[CR23] Patel V, Weiss HA, Chowdhary N, Naik S, Pednekar S, Chatterjee S (2010). Effectiveness of an intervention led by lay health counsellors for depressive and anxiety disorders in primary care in Goa, India (MANAS): A cluster randomised controlled trial. The Lancet.

[CR24] Pearson RM, Jonathan Evans D, Kounali G, Lewis J, Heron PG, Ramchandani (2013). Maternal depression during pregnancy and the postnatal period: Risks and possible mechanisms for offspring depression at age 18 years. JAMA Psychiatry.

[CR25] Sowa NA, Cholera R, Pence BW, Gaynes BN (2015). Perinatal depression in HIV-infected african women: A systematic review. Journal of Clinical Psychiatry.

[CR26] Starace F, Ammassari A, Trotta MP, Murri R, De Longis P, Izzo C (2002). Depression is a risk factor for suboptimal adherence to highly active antiretroviral therapy. Journal of Acquired Immune Deficency Syndromes.

[CR27] Stellenberg EL, Abrahams JM (2015). Prevalence of and factors influencing postnatal depression in a rural community in South Africa. African Journal of Primary Health Care & Family Medicine.

[CR28] Surkan PJ, Caitlin E, Kennedy, Kristen M, Hurley, Maureen MB (2011). Maternal depression and early childhood growth in developing countries: Systematic review and meta-analysis. Bulletin of the World Health Organization.

[CR29] Tronick E, Corrina Reck (2009). Infants of depressed mothers. Harvard Review of Psychiatry.

[CR30] van Luenen S, Garnefski N, Spinhoven P, Spaan P, Dusseldorp E, Kraaij V (2018). The benefits of psychosocial interventions for mental health in people living with HIV: A systematic review and meta-analysis. AIDS and Behavior.

[CR31] Wagner, G. J., Gwokyalya, V., Faherty, L., Akena, D., Nakigudde, J., Ngo, V., et al. (2023). *Effects of M-DEPTH Model of Depression Care on maternal HIV viral suppression and adherence to the PMTCT Care Continuum among HIV-Infected pregnant women in Uganda: Results from a cluster*. Randomized Controlled Trial at Pregnancy Completion, AIDS Behav. 10.1007/s10461-023-04014-2 PMC10386969 36907945

[CR32] Wagner GJ, Bonnie Ghosh-Dastidar E, Robinson VK, Ngo P, Glick B, Mukasa (2017). Effects of depression alleviation on ART adherence and HIV clinic attendance in Uganda, and the mediating roles of self-efficacy and motivation. AIDS and Behavior.

[CR33] Wagner, G. J., Ryan, K., McBain, D., Akena, V., Ngo, J., Nakigudde, J., Nakku (2019). Maternal depression treatment in HIV (M-DEPTH): Study protocol for a cluster randomized controlled trial, *Medicine*, 98(27). 10.1097/MD.0000000000016329 PMC6635242 31277180

[CR34] World Health Organization (2016). ‘WHO Guidelines Approved by the Guidelines Review Committee.‘ in, mhGAP Intervention Guide for Mental, Neurological and Substance Use Disorders in Non-Specialized Health Settings: Mental Health Gap Action Programme (mhGAP): Version 2.0 (World Health Organization: Geneva). 27786430

[CR35] Yonkers KA, Wisner KL, Stewart DE, Oberlander TF, Dell DL, Stotland N (2009). The management of depression during pregnancy: A report from the american Psychiatric Association and the American College of Obstetricians and Gynecologists. Obstetrics & Gynecology.

