# Supplementary material for: Treatment of Perinatal Depression and Correlates of Treatment Response Among Pregnant Women Living with HIV in Uganda
Source: Matern Child Health J. 2023 Jun 24;27(11):2017–25. doi: 10.1007/s10995-023-03741-1 (PMC10564822; doi:10.1007/s10995-023-03741-1)
Supplement: Supplementary file 1 — Supplementary Material 1 [file 10995_2023_3741_MOESM1_ESM.docx]

*Description of the usual care arm (not reported on in this study)*

Usual care for PWLWH in public ANC clinics in Uganda involves referring patients exhibiting symptoms of severe depression to a mental health specialist (often a psychiatric nurse) either at the local facility or at the District or Regional Referral Hospital. In addition, the participating ANCs offer Family Support Groups (FSG) to patients with HIV, which are led by peer mothers and nurses and follow a standard curriculum. The 24 monthly group sessions, lasting two hours each, are intended to provide psychosocial support and education around prenatal and post-partum care, including PMTCT adherence (Ugandan Ministry of Health 2017)

*Additional detail on selected measures*

***General social support*** was assessed with the modified 10-item Duke-University of North Carolina Functional Social Support Questionnaire (Broadhead et al. 1988).

***Intimate partner violence (IPV)*** was assessed using items developed for the Development and Household Survey program (Kishor and Johnson 2004). Seven items assessed types of IPV experienced over the past 6 months: emotional (e.g., accuses you of being unfaithful), controlling (e.g., insists on knowing where you are at all times), physical (e.g., ever hit, slap, kick or do anything else to hurt your physically) and sexual (e.g., physically force you to have intercourse or perform any other sexual act against your will). Respondents could answer no, yes, or don’t know. The “yes” responses were summed (range 0-7). In addition to the item that assessed the presence of physical violence, we added an item to assess the frequency of being physically hurt (with response options: not at all/sometimes/often). We derived a 3-level composite variable from the scores of these two variables: 0 = no and not at all responses to the “physical violence ever present” and frequency items; 1 = yes and sometimes to the two variables, respectively; 2 = partner often physically hurts the respondent.

***Experience of traumatic events*** during childhood (before 17 years of age) and recently (in the past 3 years) was assessed using modified versions of the Childhood Trauma Questionnaire and Recent Trauma Events Scale (Pennebaker and Susman 1988). Each of these scales assess, using yes/no responses, whether six different types of traumatic events were experienced. The scores were used to assess whether the participants experienced any traumas (as a binary variable), and the sum of traumas experienced.

***Problem-solving orientation*** (positive problem-solving orientation, negative problem-solving orientation) ***and skills*** (avoidant problem-solving styles) were assessed with the 25-item Social Problem Solving Inventory (D’Zurilla et al. 2002).

**Sources:**

Broadhead WE, Gehlbach SH, de Gruy FV, & Kaplan BHs (1988) The Duke-UNC Functional Social Support Questionnaire. Measurement of social support in family medicine patients. Med Care, 26:7, 709-723. <https://doi.org/10.1097/00005650-198807000-00006>

D’Zurilla TJ, Nezu AM, & Maydeu-Olivares A (2002) Social problem-solving inventory-revised.

Kishor S, & Johnson K. (2004). *Profiling domestic violence: A multi-country study*. MEASURE DHS+, ORC Macro.

Pennebaker JW, & Susman JRs (1988) Disclosure of traumas and psychosomatic processes. Soc Sci Med, 26:3, 327-332. <https://doi.org/10.1016/0277-9536(88)90397-8>.

Uganda Ministry of Health (2017) The role of Family Support Groups towards elimination of mother to child transmission of HIV in Uganda: an assessment report.
